# Study on the pathogenicity of *Pasteurella multocida* Type B in large-eared white rabbits

**DOI:** 10.3389/fmicb.2026.1796165

**Published:** 2026-04-10

**Authors:** Haoran Zhao, Ming Wei, Yan Feng, Xiaoxia Ren, Qi Wang, Xin Ma, Jia Wang, Junping Li, Wensheng Yao, Yizhi Zhang

**Affiliations:** 1 China Institute of Veterinary Drug Control, Beijing, China; 2 College of Veterinary Medicine, Qingdao Agricultural University, Qingdao, China

**Keywords:** bacterial load, large-eared white rabbits, *Pasteurella multocida*, pathogenicity, strain C45-2

## Abstract

To investigate the pathogenicity of *Pasteurella multocida* (Pm) Type B in large-eared white rabbits, the Pm strain C45-2 (C45-2) was used to infect rabbits. The clinical symptoms and post-mortem pathological changes of the rabbits were observed. Multiple tissues were collected from the deceased rabbits for pathological sectioning and immunohistochemical (IHC) staining. Additionally, quantitative real-time PCR (qPCR) was employed to detect the load of C45-2 in each tissue. The results showed that the minimum lethal dose (MLD) of the C45-2 to rabbits was 5 CFU. After infection, the rabbits exhibited typical Pm infection symptoms, including hemorrhage in multiple tissues and splenomegaly with dark coloration. The results of IHC and qPCR indicated that the C45-2 was more concentrated in the heart, spleen, and intestinal lymph node tissues of the infected rabbits. This study evaluated the pathogenicity of the C45-2 in infected rabbits, providing fundamental support for in-depth research on the pathogenic mechanism and vaccine development of Pm Type B.

## 1 Introduction

Bovine pasteurellosis, also known as bovine hemorrhagic septicemia, is an acute or chronic infectious disease of cattle caused by *Pasteurella multocida* (Pm) (Shivachandra et al., 2011; Wilson and Ho, 2013). This disease inflicts enormous losses on the animal husbandry industry (Govindaraj et al., 2017). For acute cases of Bovine pasteurellosis, typical symptoms include high fever, mucosal hemorrhage, and dyspnea (Cuevas et al., 2020). Post-mortem examination reveals pulmonary congestion and edema, with lung tissue appearing dark red and firm in texture; in addition, there is lymph node enlargement, a slight increase in liver size, and pinpoint-sized grayish-white necrotic foci scattered on the liver surface and cut sections (Dowling et al., 2002; Malazdrewich et al., 2001). Bovine pasteurellosis is mainly transmitted through contact. Calves and cattle herds under stress are the most susceptible (Alhamami et al., 2023; Dabo et al., 2007). There is no strict seasonality for the occurrence of this disease, but the incidence increases significantly when the temperature changes suddenly or under humid and sweltering conditions, and it often presents as an endemic (Mo et al., 2024). Literature related to the epidemiology of Pm shows that the mortality of hemorrhagic septicemia among cattle and buffaloes increased in 2017–2019 (Almoheer et al., 2022). In 2024, the positive rate of Pm in nasal swabs of beef cattle from 24 large-scale cattle farms across 8 districts in Ningxia was 20.2%. The dominant serotypes were Type A (57%) and Type B (38%), with cattle aged 3–8 months being the high-incidence group (Guo et al., 2024). A survey conducted from 2022 to 2024 covering 54 pastures in 16 regions across multiple provinces of china revealed that the positive detection rate of this disease was 10.32%, and it was often associated with mixed infection with *Mannheimia haemolytica* (Mh)(Yu et al., 2025; Zecchinon et al., 2005). The mortality rate of acute cases can exceed 80%, while cattle that recover develop strong specific immunity. Currently, the prevention of bovine pasteurellosis mainly relies on immunization, which can significantly reduce the risk of infection (Ahmad et al., 2014; Chandrasekaran et al., 1994; Fegan et al., 2024; Mostaan et al., 2020; Verma and Jaiswal, 1998). Existing domestic vaccine products for bovine pasteurellosis include inactivated vaccines prepared from capsular Type B strains, which can prevent hemorrhagic septicemia; there are also bivalent inactivated vaccines prepared from Type A and Type B strains, which can prevent the occurrence of mixed infections (Islam et al., 2023). Vaccines for the prevention of bovine pasteurellosis available in foreign markets are predominantly monovalent inactivated formulations derived from serogroup B Pm, together with bivalent inactivated vaccines that confer concurrent protection against both Pm and Mh infections. When cattle are infected with this disease, sensitive drugs such as *β*-lactams and fluoroquinolones can be used to control the proliferation of Pm (Coates and Hu, 2007; Jamali et al., 2014; Kehrenberg et al., 2001). Drugs like flunixin meglumine can be administered intramuscularly to relieve fever and inflammatory reactions, and glucose normal saline can be injected intravenously to supplement water and energy.

The Pm strain C45-2 (C45-2) was isolated from cattle with hemorrhagic septicemia in Qinghai Province, belonging to serotype B, and is preserved in the National Veterinary Microbial Culture Collection Center. In accordance with the current Chinese Veterinary Pharmacopoeia, C45-2 is used in China as the strain for potency testing of the serotype B component in inactivated vaccines against bovine pasteurellosis. It is applicable for both the immunization-challenge test in target animals (cattle) and the immunization-challenge test in surrogate animals (rabbits).

This study aims to investigate the pathogenicity of strain C45-2 in large-eared white rabbits. Since rabbits are sensitive to Pm serotype B, experiments including *in vivo* virulence determination, hematological detection, pathological dissection, histological observation, and measurement of Pm load in tissues were conducted. This study provides fundamental support for in-depth understanding of the pathogenesis of strain C45-2 (Hodgson et al., 2013). Furthermore, it offers a scientific basis for using laboratory rabbits as a surrogate animal model to evaluate the immune efficacy of inactivated vaccines against bovine pasteurellosis.

## 2 Materials and methods

### 2.1 Ethics statements

All animal studies were performed in accordance with the Laboratory Animal Ethics Committee of China Institute of Veterinary Drug Control and followed the International Guiding Principles for Biomedical Research Involving Animals.

### 2.2 Reagents

Modified Martin Agar Medium, Martin Broth Medium, and physiological saline were all purchased from Beijing Zhonghai Biotechnology Co., Ltd.; and newborn calf serum was purchased from Sijiqing Biotechnology Co., Ltd.

### 2.3 Experimental animals

Conventional-grade Japanese large-eared white rabbits weighing 1,500–2,000 g were purchased from Beijing Longan Experimental Animal Breeding Center.

### 2.4 Bacteria strains and culture conditions

The Pm strain C45-2 (CVCC44502) was provided by the National Veterinary Microbial Culture Collection Center. One vial of lyophilized C45-2 was rehydrated with 0.5 mL of Martin Broth. A small amount of the bacterial suspension was dipped with an inoculating loop and streaked onto a modified Martin Agar plate containing 0.1% lysed whole blood and 4% newborn calf serum, followed by incubation at 37 °C for 18 h. Typical colonies on the plate—characterized by a smooth surface and pale blue color; under low-magnification microscopy (observed at a 45° refractive angle), the colonies showed a rough structure, neat edges, blue-green iridescence, and a reddish-yellow light band at the edge—were picked and inoculated into Martin Broth medium containing 0.1% lysed whole blood and 4% newborn calf serum. The inoculated medium was statically incubated at 37 °C for 24 h until the broth culture became turbid.

### 2.5 Viable bacterial counting

An appropriate amount of bacterial suspension was subjected to 10-fold serial dilution. Suitable dilution levels were selected based on the bacterial count. For each dilution level, 0.1 mL of the bacterial suspension was aspirated and inoculated onto 3 modified Martin Agar plates. A sterile spreader was used to spread the bacterial suspension evenly on the plates. The plates were placed upright in a 37 °C incubator for 30 min, then inverted for continued incubation. After 24 h, the number of colonies on each plate was counted. The concentration of the original bacterial suspension (CFU/mL) was calculated using the formula: (X₁ + X₂ + X₃)/3 × 10 × serial dilution factor, where X₁, X₂, and X₃ represent the number of colonies on each of the three plates.

### 2.6 Cryopreservation and quantification of C45-2

To the cultured Pm Martin Broth culture, glycerol was added to a final concentration of 20%. The mixture was aliquoted into 15 mL centrifuge tubes at 3 mL per tube and cryopreserved at below −70 °C. Two days before the challenge, the cryopreserved C45-2 strain was retrieved for viable bacterial counting.

### 2.7 Virulence assay of C45-2 in rabbits

Conventional-grade Japanese large-eared white rabbits were reared in an ABSL-2 (Animal Biosafety Level 2) negative-pressure animal room. Based on the viable bacterial counting results, the C45-2 bacterial suspension was diluted to concentrations of 1 CFU/mL, 3 CFU/mL, and 5 CFU/mL, respectively. Each rabbit was subcutaneously injected with 1 mL of the diluted bacterial suspension, with 2 rabbits assigned to each dose group. The survival status of the rabbits was observed and recorded daily, and the minimum lethal dose (MLD) of C45-2 for rabbits was calculated.

### 2.8 Sample collection

At 24 h post-challenge, sterile blood samples were collected from the rabbits. One tube of blood was used for serum separation, and the separated serum was sent to Beijing Zhongke Gene Technology Co., Ltd. for liver function testing using the diffraction grating spectroscopy method. The other tube of blood was subjected to anticoagulation treatment before being used for routine blood testing. The rabbits were then subjected to necropsy to observe the pathological changes in various organs and tissues. Tissues including the liver, heart, spleen, lung, kidney, and intestinal lymph nodes were collected, fixed in 4% paraformaldehyde, and subsequently stained with hematoxylin and eosin (H&E) to observe histopathological changes.

### 2.9 IHC detection of rabbit tissues

Tissues (liver, heart, spleen, lung, kidney, and intestinal lymph nodes) were collected from deceased rabbits. Immunohistochemical staining was performed using bovine-derived Pm-positive serum as the primary antibody to compare the bacterial infection status across different rabbit tissues. For each tissue section, 3 non-overlapping fields of view were randomly selected and imaged under 250× magnification. Image analysis was performed in a blinded manner with a consistent threshold applied uniformly. Using Image-Pro Plus 6.0 in Basic mode, background intensity was set, and IOD was selected as the quantitative parameter. Positively stained areas were marked and calculated via the Count/Size function, and the mean value was used as the quantitative result for each sample.

### 2.10 Quantification of C45-2 load in rabbit tissues by qPCR

Under sterile conditions, tissues (liver, heart, spleen, lung, kidney, and mesenteric lymph nodes) were collected from rabbits. After quick-freezing in liquid nitrogen, DNA was extracted from each tissue. Subsequently, the C45-2 load in each tissue was determined using the Pm qPCR method previously established in the laboratory. For quantification, triplicate PCR reactions were performed for each tissue sample. The average Ct value (Ct adv) of the three replicates was substituted into the linear regression equation between Pm copy number and Ct value: *Y* = −3.5663lg(X) + 42.296. Samples with a Ct value ≤ 37 were considered positive, whereas those with a Ct value > 37 were regarded as negative or non-detectable.

### 2.11 Statistical analysis

The qPCR results were analyzed using ABI Real-Time PCR Software v2.4, and the average Ct value of triplicate wells was used to calculate the copy number of Pm. For IHC analysis, the mean optical density (IOD Sum/Area Sum) was used as the index to evaluate bacterial burden. Data summary and analysis were performed using GraphPad Prism 6 software.

## 3 Results

### 3.1 Determination of the virulence of C45-2 in rabbits

Based on the results of viable bacterial counting, the diluted C45-2 bacterial suspension was subcutaneously injected into rabbits, resulting in a final injection concentration of 1 CFU per rabbit, 3 CFU per rabbit, and 5 CFU per rabbit. The death of rabbits was recorded (Table 1). The results showed that the minimum lethal dose of C45-2 for Japanese large-eared white rabbits was 5 CFU.

**Table 1:** Results of virulence determination of C45-2 in rabbits.

Strain	Number of rabbits	Injection dose (individual)	Number of rabbit deaths within 8 days post-challenge (individuals)								Results
			Day 1	Day 2	Day 3	Day 4	Day 5	Day 6	Day 7	Day 8	
C45-2	2	0 CFU	0	0	0	0	0	0	0	0	0/2 deaths
	2	1 CFU	0	1	0	0	0	0	0	0	1/2 deaths
	2	3 CFU	0	1	0	0	0	0	0	0	1/2 deaths
	2	5 CFU	1	1	/	/	/	/	/	/	2/2 deaths

### 3.2 Detection of blood indicators in rabbits infected with C45-2

Twenty-four hours after infection, liver function tests (Table 2) and routine blood tests (Table 3) were performed on rabbits in each group. In the results, the rabbits numbered 1 CFU-1, 3 CFU-1, 5 CFU-1, and 5 CFU-2 were the deceased ones. Among them, the rabbit numbered 5 CFU-2 died within 24 h post-infection. The liver function test results showed that this rabbit had an abnormal alkaline phosphatase (ALP) level, and the routine blood test results indicated that it had low counts of white blood cells (WBCs) and platelets (PLTs). The rabbits numbered 1 CFU-1, 3 CFU-1, and 5 CFU-1 died within 24–48 h post-infection, all of which exhibited ALP levels. In addition, a general decrease in WBCs and PLTs counts was observed in their routine blood tests.

**Table 2:** Results of liver function tests in rabbits infected with C45-2.

Test items	Abbreviations	Units	Reference range	Original numbers							
				Control-1	Control-2	1 CFU-1	1 CFU-2	3 CFU-1	3 CFU-2	5 CFU-1	5 CFU-2
Albumin	ALB	μmol/L	22–40	34.58	37.72	39.34	37.49	42.17	34.71	41.9	37.39
Total bile acid	TBA	U/L	/	10.82	12.28	12.06	8.75	15.7	29.62	50.22	19.14
Alanine aminotransferase	ALT	U/L	5–106	47.03	27.56	68.55	26.56	51.54	29.31	81.9	71.86
Aspartate aminotransferase	AST	U/L	0–98	51.86	15.13	18.52	14.85	32.99	126.79	117.2	25.6
Alkaline phosphatase	ALP	U/L	20–145	120.76	59.6	177.92	55.92	197.49	115.9	401.36	174.12
Total bilirubin	T-BIL	U/L	0–14	0.25	0.88	0.5	0.71	0.53	0.66	3.26	0.58
*γ*-glutamyl transferase	*γ*-GGT	μmol/L	0–14	7.59	9.8	10.07	10.05	9.67	8.33	37.6	8.25

The measured data with an underline indicates that the value is abnormal and not within the reference range. Units are provided by the analyzer and vary depending on the detection method.

**Table 3:** Results of routine blood tests in rabbits infected with strain C45-2.

Test items	Abbreviations	Units	Reference range	Original numbers							
				Control-1	Control-2	1 CFU-1	1 CFU-2	3 CFU-1	3 CFU-2	5 CFU-1	5 CFU-2
White blood cell count	WBC	10^9^/L	3–12.5	10.18	5.79	2.37	7.06	2.91	11.48	0.76	1.91
Neutrophil percentage	NEU	%	24–62	43.6	44.6	54	35.3	42.6	55.2	8.2	54.2
Lymphocyte percentage	Lym	%	15–64	47.4	44.1	39	55	50.6	35.9	64.6	35.5
Monocyte percentage	MON	%	0–20	5.7	5.3	5.5	6.1	4.2	5.5	9.3	7.6
Basophil percentage	BAS	%	0–8	1.3	3	0.7	1.9	1.8	2.6	0	2.5
Platelet count	PLT	10^9^/L	200–800	237	246	127	422	216	421	16	43
Hematocrit	HCT	%	25–42	36.5	32.7	35.4	40.5	35.8	29.6	33.7	37.6
Red blood cell count	RBC	10^12^/L	4.15–6.5	5.68	4.99	6.05	6.27	6.17	4.55	5.33	6.14
Hemoglobin	Hb	g/L	85–165	119	108	114	130	115	95	110	129

The measured data with an underline indicates that the value is abnormal and not within the reference range.

### 3.3 Clinical symptoms and pathological changes of rabbits infected with C45-2

**Figure 1:**
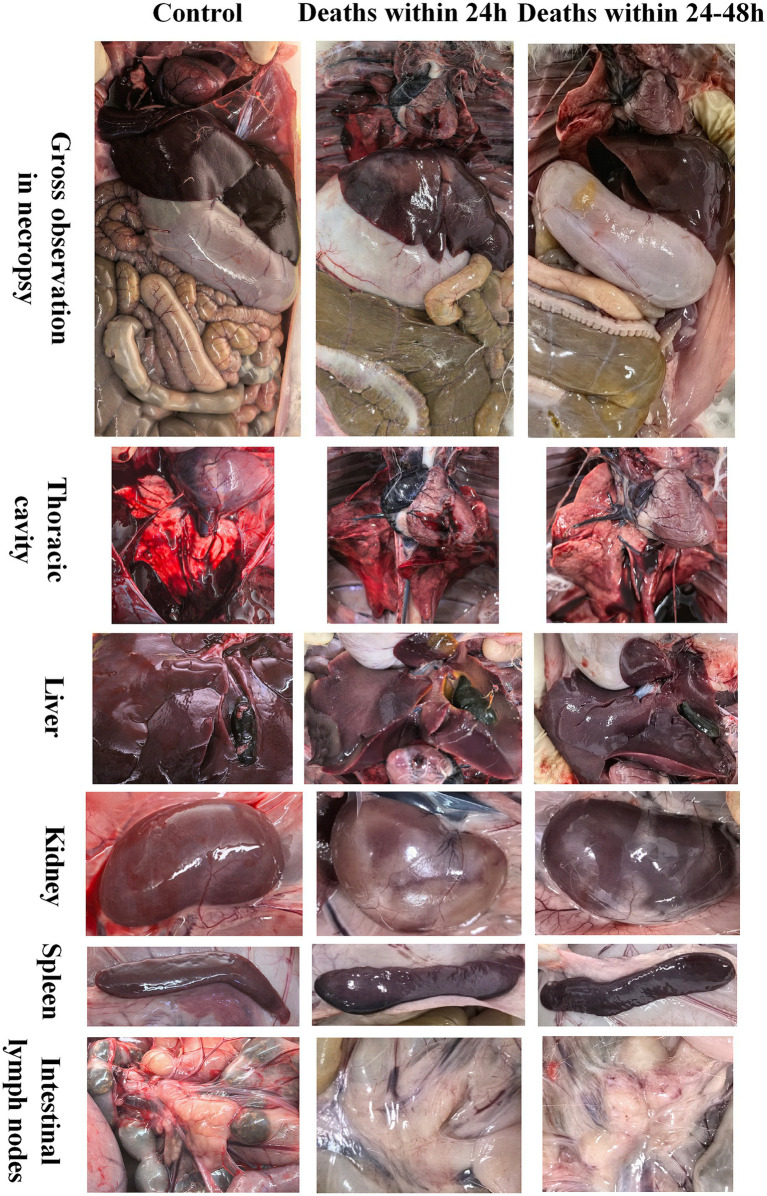
Post-mortem examination and tissue lesion findings in rabbits infected with C45-2.

Some rabbits began to show symptoms such as listlessness, anorexia, difficulty standing, and trembling 12 h after challenge. Within 24 h post-infection, one rabbit in the 5 CFU group died; between 24 and 48 h post-infection, one rabbit died in each of the three dose groups. No further rabbit deaths occurred after 48 h post-infection. Necropsies were performed on both dead and surviving rabbits. Rabbits that died after C45-2 infection exhibited the following pathological changes: hepatic hemorrhage with pale edges, reduced myocardial elasticity, pulmonary hemorrhage, splenomegaly (enlarged spleen) with dark edges, intestinal flatulence, and dark red coloration in some lymph nodes. Among these, rabbits that died within 24 h post-infection showed more severe pulmonary hemorrhage, while those that died between 24 and 48 h post-infection had more severe splenic hemorrhage (Figure 1).

### 3.4 Histopathological changes in rabbits infected with C45-2

**Figure 2:**
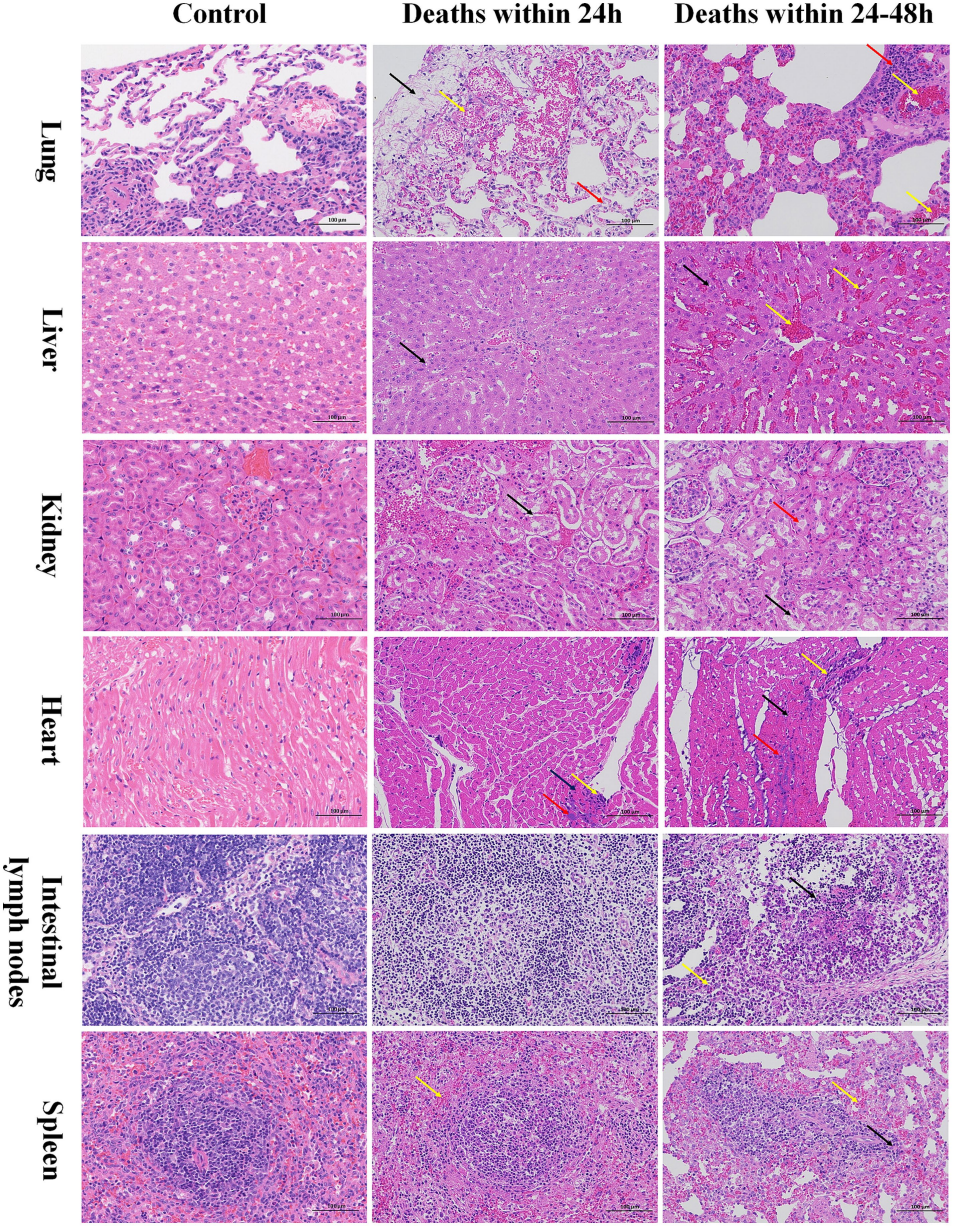
Histopathological changes in various tissues of rabbits infected with C45-2 (200×).

H&E staining was performed on various rabbit tissues, and the results are shown below (Figure 2). All lung tissues of infected rabbits exhibited significant capillary congestion (yellow arrows). For rabbits that died within 24 h post-infection, their lung tissues showed local edema of the visceral pleura (black arrows), extensive cytoplasmic vacuolization of alveolar wall epithelial cells (red arrows). There was focal lymphocytic infiltration was observed around numerous blood vessels and bronchioles (red arrows) from rabbits that died between 24 and 48 h post-infection. In the liver tissues of rabbits that died within 24 h, a small number of hepatocytes showed mild steatosis locally (black arrows). In contrast, in the liver tissues of rabbits that died between 24 and 48 h, extensive congestion was observed in the hepatic veins and sinusoids (yellow arrows); mild steatosis of hepatocytes was seen around multiple venous vessels. In the renal tissues of rabbits that died within 24 h, extensive detachment of renal tubular epithelial cells was observed (black arrows). In the renal tissues of rabbits that died between 24 and 48 h, a small amount of renal tubular epithelial cell detachment was seen locally, and eosinophilic substances were present in the lumens of some renal tubules (red arrows). The cardiac tissue changes in infected rabbits were consistent: localized spotty myocardial fiber necrosis was observed in all cases (black arrows), accompanied by karyopyknosis and hyperchromatism. Additionally, spotty lymphocytic infiltration was present (yellow arrows), along with a small number of basophilic masses (red arrows). In the lymph nodes of rabbits that died within 24 h, no obvious abnormalities were observed. In the lymph nodes that died between 24 and 48 h, extensive lymphocyte necrosis and karyorrhexis were seen in local lymphoid follicles within the cortex of lymphoid tissues (black arrows), accompanied by a small amount of granulocyte infiltration (yellow arrows). In the spleens of infected rabbits, the boundary between the red pulp and white pulp was indistinct, and mild congestion was widely observed in the red pulp (yellow arrows). In the spleens that died between 24 and 48 h, a large number of splenic nodules showed deformation and reduced volume (black arrows).

### 3.5 IHC detection in various tissues of rabbits infected with C45-2

**Figure 3:**
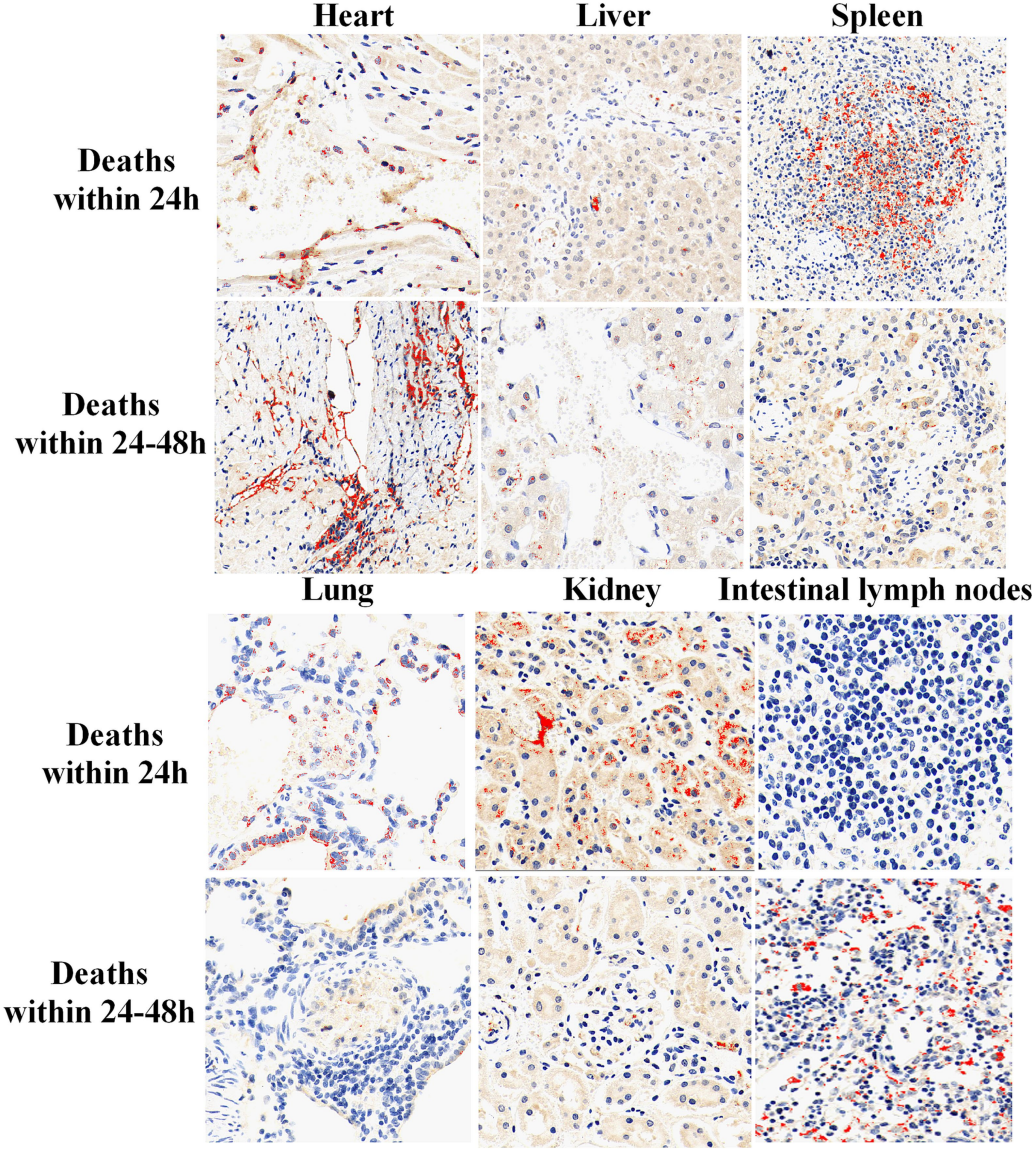
IHC detection results of various tissues in rabbits infected with C45-2 (250×).

A variety of tissues from rabbits that died within 24 h and between 24 and 48 h after C45-2 infection were collected for IHC. The images were quantitatively analyzed using Image-Pro Plus 6.0 software. A specific color threshold was set to accurately identify the positive staining areas. The sum of optical density values (IOD Sum) of all pixels in the positive staining areas within the image area of the infected tissue and the total number of pixels in the positive staining areas in the image (Area Sum) were analyzed. The average optical density (IOD Sum/Area Sum) was used as an indicator to analyze the bacterial count (Table 4), so as to quantify the load and distribution of Pm in each tissue. The results showed that the loads of C45-2 were high in the spleen and kidney of rabbits that died within 24 h, and high loads were detected in the heart and intestinal lymph nodes of rabbits that died between 24 and 48 h (Figure 3).

**Table 4:** Detection of Pm load in various tissues of rabbits infected with C45-2 by qPCR.

Group	Name	Ct			Ct adv	Number of copies	IOD Sum/Area Sum
Deaths within 24 h	Heart	21.18	21.26	21.32	21.25	7.9511 × 10^5^	0.19
	Liver	22.30	22.20	22.11	22.20	4.3057 × 10^5^	0.13
	Spleen	7.50	6.84	9.71	8.02	4.0927 × 10^9^	0.24
	Lung	8.83	8.62	10.23	9.22	1.8738 × 10^9^	0.20
	Kidney	8.70	8.54	9.10	8.78	2.4623 × 10^9^	0.22
	Lymph nodes	ND	ND	ND	ND	ND	0
Deaths within 24–48 h	Heart	17.20	17.27	17.18	17.21	1.0772 × 10^7^	0.38
	Liver	21.38	21.31	21.58	21.42	7.1246 × 10^5^	0.14
	Spleen	19.98	19.93	19.97	19.96	1.8326 × 10^6^	0.18
	Lung	23.34	22.25	21.41	22.33	3.9591 × 10^5^	0.12
	Kidney	21.78	21.89	23.92	22.53	3.4869 × 10^5^	0.11
	Lymph nodes	16.62	16.98	16.62	16.74	1.4654 × 10^7^	0.36

Samples with a Ct value > 37 were regarded as non-detectable (ND).

### 3.6 Detection of Pm load in various tissues of C45-2 infected rabbits by qPCR

**Figure 4:**
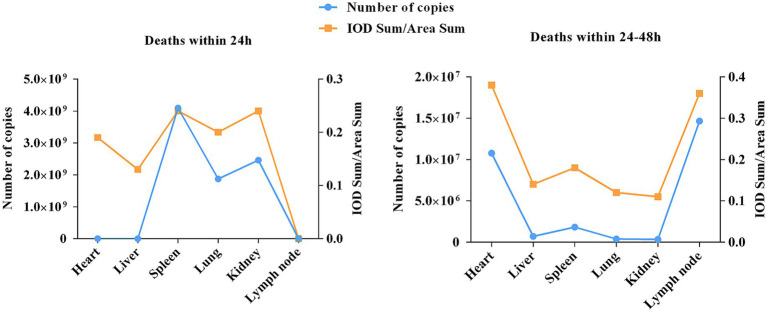
Comparison of Pm load results in various tissues of rabbits infected with C45-2 determined by IHC and qPCR. Number of copies represents the Pm load analyzed by qPCR detection, while IOD Sum/Area Sum represents the quantitative index of Pm analyzed by IHC detection.

According to the linear relationship *Y* = −3.5663 lg(X) + 42.296 between Pm copy number and Ct value in the absolute fluorescent quantitative PCR method established by Wang et al., the Pm load in various tissues of rabbits was determined (Table 4). The results showed that the load of C45-2 were high in the spleen, kidney and lung of rabbits that died within 24 h, and high loads were detected in the intestinal lymph nodes and heart of rabbits that died between 24 and 48 h, which was similar to the trend of IHC results (Figure 4). This result suggests that the tissue distribution of C45-2 varies at different stages of infection.

## 4 Discussion

In this study, Japanese large-eared white rabbits were used as the experimental animal model. Through multi-dimensional experiments including *in vivo* virulence determination, hematological testing, pathological necropsy, histological observation, and detection of Pm load in tissues, the pathogenicity of C45-2 to rabbits and the pathophysiological changes after infection were systematically investigated. The results (see Table 1) showed that the minimum lethal dose of C45-2 to large-eared white rabbits was 5 CFU. After infection, the rabbits exhibited typical Pm infection symptoms such as hemorrhage in multiple tissues, splenomegaly with dark color, and myocardial damage.

The results of hematological tests at 24 h post-C45-2 infection (see Tables 2, 3) showed that all dead rabbits exhibited abnormalities in alkaline phosphatase (ALP), along with a significant decrease in the counts of white blood cells (WBCs) and platelets (PLTs). This change was highly correlated with the degree of pathological damage (Periasamy et al., 2018). ALP is mainly derived from tissues such as the liver and bones. An abnormal increase in its activity usually indicates hepatocyte damage or biliary system dysfunction. Combined with the manifestations of hepatic hemorrhage and hepatocellular steatosis identified in histology, it is confirmed that C45-2 infection can directly cause liver function damage. The decrease in WBCs count may be related to the destruction of immune cells by hemolysins, cytotoxins, and other substances produced by C45-2. The reduction in PLTs may result from coagulation dysfunction caused by vascular endothelial damage, or insufficient platelet production due to damage to hematopoiesis-related tissues such as the spleen. These two changes together weaken the body’s immune defense and hemostatic functions, further exacerbating the pathological process after infection. No significant abnormalities were found in the blood indicators of rabbits that survived the infection, suggesting that the body may have resisted the damage from low-dose infection through its own immune regulation, or the proliferation of the strain in the body was restricted.

The results of necropsy (see Figure 1) showed that after C45-2 infection, the main damaged organs of rabbits were concentrated in the lungs, heart, liver, spleen, and intestinal lymph nodes, presenting with hemorrhage, edema, and tissue degeneration. However, there were differences in the characteristics of tissue damage among rabbits that died at different time periods: rabbits that died within 24 h had relatively severe pulmonary hemorrhage, while rabbits that died between 24 and 48 h had relatively severe splenic hemorrhage. Histological observations (see Figure 2) further revealed the details of tissue damage: In the lung tissues of rabbits that died within 24 h, vacuolization of alveolar epithelial cells, capillary congestion, and spotty necrosis of myocardial fibers were observed; however, no obvious abnormalities were found in either the cortical or medullary regions of the lymph nodes. This suggests that during the acute infection phase, C45-2 first invades parenchymal organs such as the lungs, triggering acute tissue damage. In rabbits that died between 24 and 48 h post-infection, focal lymphocytic infiltration was observed around the blood vessels and bronchioles in lung tissues, along with extensive congestion in hepatic sinusoids, significant lymphocyte necrosis in local lymphoid follicles of the intestine, and massive deformation of splenic nodules. This indicates that as the infection duration extends, the damage gradually spreads to immune-related tissues, and the impairment of immune cells may further reduce the body’s ability to clear the pathogen.

The detection results of Pm load in infected rabbit tissues (see Figure 4) indicated that during acute infection, C45-2 was mainly concentrated in the spleen, heart, and intestinal lymph node tissues of rabbits. The tissue distribution of bacterial load of strain C45 2 in rabbits showed obvious time dependence and organ tropism, and such differences were closely related to the invasive characteristics of the strain, infection process, physiological features of tissues, and host immune response. This study only analyzed rabbits that died within 24 h and between 24 and 48 h post-infection, without dynamic monitoring of surviving rabbits or continuous time-point tracking of tissue bacterial load, making it difficult to fully reveal the proliferation and dissemination dynamics of strain C45 2 in rabbits. In addition, the natural target host of strain C45 2 is cattle, and its natural transmission occurs mainly via the respiratory tract, whereas this study used subcutaneous injection to challenge rabbits. Therefore, this model cannot fully simulate the bacterial colonization and dissemination patterns under natural infection routes, and can only provide evidence for using rabbit challenge experiments as an alternative method for efficacy testing of inactivated vaccines against bovine pasteurellosis.

This study preliminarily explored the pathogenicity of C45-2 to large-eared white rabbits, identified the pathophysiological changes and tissue distribution of C45-2 in rabbits after infection, and determined the minimum lethal dose of this strain to Japanese large-eared white rabbits. This study provides theoretical support for the research on the virulence and pathogenic mechanism of strain C45-2. Moreover, it provides a scientific basis for using rabbit challenge experiments as an alternative method for efficacy testing of inactivated vaccines against bovine pasteurellosis.

## Data Availability

The original contributions presented in the study are included in the article/supplementary material, further inquiries can be directed to the corresponding author.
